# CRISPR/Cas9-induced Targeted Mutagenesis and Gene Replacement to Generate Long-shelf Life Tomato Lines

**DOI:** 10.1038/s41598-017-12262-1

**Published:** 2017-09-19

**Authors:** Qing-hui Yu, Baike Wang, Ning Li, Yaping Tang, Shengbao Yang, Tao Yang, Juan Xu, Chunmiao Guo, Peng Yan, Qiang Wang, Patiguli Asmutola

**Affiliations:** Institute of Horticulture, Xinjiang Academy of Agricultural Science, Urumqi, 830091 China

## Abstract

Quickly and precisely gain genetically enhanced breeding elites with value-adding performance traits is desired by the crop breeders all the time. The present of gene editing technologies, especially the CRISPR/Cas9 system with the capacities of efficiency, versatility and multiplexing provides a reasonable expectation towards breeding goals. For exploiting possible application to accelerate the speed of process at breeding by CRISPR/Cas9 technology, in this study, the *Agrobacterium tumefaciens*-mediated CRISPR/Cas9 system transformation method was used for obtaining tomato *ALC* gene mutagenesis and replacement, in absence and presence of the homologous repair template. The average mutation frequency (72.73%) and low replacement efficiency (7.69%) were achieved in T_0_ transgenic plants respectively. None of homozygous mutation was detected in T_0_ transgenic plants, but one plant carry the heterozygous genes (*Cas9*/*-*ALC*/*alc*) was stably transmitted to T_1_ generations for segregation and genotyping. Finally, the desired *alc* homozygous mutants without T-DNA insertion (*/*-*alc*/*alc*) in T_1_ generations were acquired and further confirmed by genotype and phenotype characterization, with highlight of excellent storage performance, thus the recessive homozygous breeding elites with the character of long-shelf life were generated. Our results support that CRISPR/Cas9-induced gene replacement via HDR provides a valuable method for breeding elite innovation in tomato.

## Introduction

Breeding technologies are critical for improving crop production in our changing world with an exponentially growing population and in the face of extreme environmental change^1^. MAS and transgenic breeding have become the two major procedures of modern plant breeding schemes. Despite the enormous advantages, there are still the limitations. The backcrossing and MAS processes are laborious, time-consuming and besides unavoidable introgression of closely-linked undesirable traits from donor organisms. Although these defects can be overcome through the latter, transgenic breeding still presents the major denunciation about the random gene insertions throughout the genome and potential disruption of endogenous genes function, remarkably, with the advent of gene editing technologies, especially the game-changing technology–the CRISPR/Cas9 system, offering an extremely efficient and simple customization process. In principle, it can target any site of interest as well as the most cost effective cloning^2^, even entry-level researchers can successfully implement, appears to be a promising tool for revolutionizing basic research and plant breeding^3^. Gene editing relies on sequence-specific nucleases (SSNs) to trigger DNA double-strand breaks (DSBs) at a desired location within the genome. The DSBs are repaired by either non-homologous end joining (NHEJ) or homology-directed repair (HDR)^4^. The former which usually leads to various modifications of the targeted sequence, such as deletions or insertions. Unlikely, native genes can be replaced or corrected by the latter when a homologous DNA donor repair template to the targeted gene is present. From the perspective of crop breeders, the HDR is more attractive than NHEJ, which can precisely integrate the desired gene with the important agricultural trait from gene pool into a target site within a genome intentionally by replacements. This is paying the way for straightforward and accelerated introgression a single gene, even pyramiding some elite genes into the breeding lines, thus provide a high-efficiency and promising strategy for plant breeding.

The long-shelf life is a critical trait for the quality of fleshy fruit, and it is one of the major objectives in breeding programs as it influences fruit marketability for both farmer’s and consumer’s perception. Tomato (*Solanum lycopersicum*) has been mostly studied as classic climacteric model species with fleshy fruits to unravel the molecular basis of fruit softening. There are several naturally occurring mutations with the long-shelf life properties that affect the ripening of fruit, such as *Nr* (Never Ripe)^5^, *alc* (alcobaca)^6^ which synonymous with the DFD (delayed fruit deterioration) mutation^7–9^ *rin* (ripening inhibitor)^10^, *nor* (non-ripening)^11^ and *Cnr* (colorless non-ripening)^12^, and these genes have been cloned and studied at the molecular level (NM_001246965, FJ404469, NM_001247047, NM_001319308 and NM_001319308). In addition the mutations *rin*, *nor* and *alc* have been used for long-shelf life breeding programs with the heterosis for yield^13,14^. Although theses mutations negatively affect organoleptic quality^15,16^, *alc* mutation seems more appropriate than *rin* and *nor* in the breeding of long-shelf life varieties with a low negative impact on fruit quality, particularly better skin color, aroma properties and resistance to bacterial disease^9,17^. The molecular basis of the *alc* mutation is the replacement of thymine by adenine in position 317 of the coding sequence of the NOR gene, this one base pair mutation is a nonsynonymous amino acid change, namely the valin (Val) was replaced by the aspartic acid (Asp). Therefore, confirming it is an allele of *nor* gene^8^.

To confirm the feasibility of pre-determined locus mutagenesis, targeted gene replacement to facilitate rapid improving shelf life of breeding elites in tomato. In this study, a CRISPR/Cas9 system suitable for plant genome editing was used to determine efficiencies in the absence and presence of the dsDNA donor repair template. The results show that both pre-determined locus mutagenesis and targeted gene replacement events can be occurred respectively, and the allele of *ALC* was replaced by *alc* gene via HDR repair pathway. The heterozygote plants in T_0_ generations, and the homozygote plants in subsequent T_1_ generations were generated. The agronomic and morphological analyses of homozygote plants were consistent with long-shelf life conferred by substitution of the *alc* gene. The date presents here hold great potential for tomato genetics and breeding.

## Results

### CRISPR-mediated *SLALC* Mutagenesis in T_0_ Transgenic Tomato

**Figure 1:**
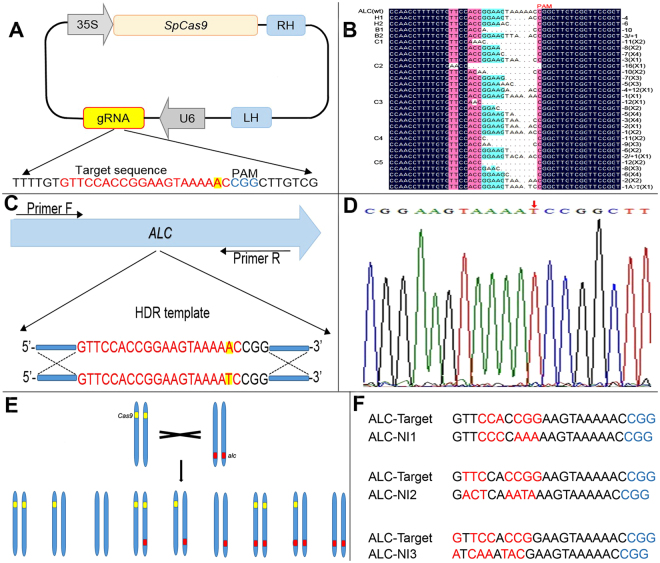
Tomato gene editing system mediated by CRISPR/Cas9. (**A**) The schematic description of CRISPR/Cas9-sgRNA expression cassette: *Sp*Cas9 is controlled by a CaMV *35S* promoter, the sgRNA is expressed by the *AtU6* promoter; Red letters mark the target sequence of *ALC* target site, blue letters mark the PAM (the protospacer adjacent motif), yellow highlighted ‘A’ marks the mutational site of *ALC*/*alc* gene. (**B**) The sequenced targeted InDel mutation of *ALC* gene. 24 clones of the each PCR amplicon were picked and sequenced; “H” means Heterozygote, “B” means Biallele, “C” means Chimera. (**C**) Strategy of Homologous-directed Repair: Red letters mark the target sequence recognized by cognate sgRNA, yellow highlighted letters mark the replacement and be replacement. (**D**) Sequencing results of repair fragment through HDR: Red arrow points to the base which is the repair base through HDR. (**E**) The gene segregation in T_1_ populations of a single plant (*Cas9*/*-*ALC*/*alc*) which from T_0_ lines. (**F**) The potential off-target sites of *ALC* gene detected in tomato genome: ALC-Target marks the target sequence, ALC-NI (Nearly Identical sequences) mark the potential off-target sequences; Blue letters marks the PAM sequence, red letters mark the unmatched bases.

To detect the Cas9 endonuclease with a sgRNA can generate DSBs and alter *ALC* gene sequences through error-prone NHEJ repair, a codon optimized version of Cas9 is controlled by a CaMV *35S* promoter, the sgRNA is expressed by the *AtU6* promoter (Supplementary Fig. 1), and both elements are constructed to the same T-DNA vector of pCAM1301, the sgRNA was designed with the guide sequence matching a 20-bp region alongside the protospacer-adjacent motif sequence (PAM) within the *ALC* gene (Fig. 1A).

The synthetic plasmid was transfected into hypocotyls of tomato M82 through *Agrobacterium*-mediated stable transformation, total 11 independent T_0_ lines with the Cas9 gene transgenic positive reaction were obtained (Supplementary Fig. 2). The genomic DNA was extracted from the leaf of each transgenic plant, following the PCR was performed with the designed primers flanking the target region (Supplementary Table 1 and Supplementary Fig. 3), and then 24 clones of the each PCR amplicon were picked and sequenced, eventually total 264 clones were sequenced and analyzed (Fig. 1B). The results showed that there were three non-mutated lines (designated as WT) and the rest of 8 lines carried mutations, determined the mutation rate was 72.73% (8/11). If there is just one mutant type and the ratio of the mutant type to the wild type is 1:1, this mutant type is regarded as the heterozygous. As for the allele, if there are two mutant types and no wild type, and the ratio of the two mutant types is 1:1. If there are more than one mutant types and wild type still could be found. The ratios of these types are disproportionate, then this mutant type is regarded as the chimeric. After analysis of the genotypes of mutants indicated that there were 2 heterozygous, one biallelic and 5 chimeric mutants, but none homozygous mutant was detected (Table 1). The types of mutants were also analyzed, most the mutation varieties we obtained were 1- to 16-bp deletions (122), the rest of four types including two mutants were 1-bp insertion accompany with 2- to 3-bp deletions, one unique mutant was 4-bp deletions accompany with 12-bp insertions, and one mutant was 1-bp deletion with one nucleotide replacement (T → A) (Fig. 1B).

**Table 1:** Mutation of *ALC* gene in tomato T_0_ generation.

Target gene	N0. of detected lines	Zygosity				
		Homozygote	Heterozygote	Biallele	Chimera	WT
*ALC*	11	0	2(18.18%)	1(9.09%)	5(45.45%)	3(27.27%)

### Targeted Gene Replacement in T_0_ Transgenic Tomato through HDR Pathway

In order to realize the accurate replacement of *alc* for *ALC* gene in the target site, a double strands DNA donor template was constructed and inserted into the vector downstream the sgRNA. For the reason only one base substitution (T → A) was desired, the 23-bp sequence (20-bp sgRNA and 3-bp PAM) was chosen as the middle core sequence of the donor fragment, this middle core sequence was flanked with the 235-bp left arm and the 450-bp right arm, which were identical to the stretches of targeted sequences (Fig. 1C and Supplementary Fig. 4). The synthetic plasmid which contains the Cas9/sgRNA and ds-DNA fragment donor was transfected into hypocotyls through the same *Agrobacterium*-mediated transformation method. Due to the low efficiency of HDR events in most plants since the predominant repair pathway in somatic plant cells is NHEJ, the large-scale screening efforts were required to ensure the occurrence of HDR-mediated replacement, therefore we induced callus derived from hypocotyls, and proliferated them as much as possible to generate more transgenic plants, finally, we got 26 positive transgenic plants, and then the PCR products were directly cloned and sequenced(Supplementary Table 1), 10 clones of the each PCR amplicon were picked and total 260 clones were sequenced. The results showed that HDR-mediated repair events were occurred in 2 transgenic plants, including 1 plant with the heterozygous allele and 1 plant with chimeric allele respectively, which were confirmed by sequencing. However, none repaired homozygous allele was detected and the rest of 24 plants weren’t edited and designated as WT or mutant, thus the replacement efficiency calculated as 7.69% (2/26) (Table 2 and Fig. 1D).

**Table 2:** Homology-directed repair of the target site of *ALC* gene in tomato T_0_ generation.

Target gene	Expectation repaired gene	N0. of examed lines	Zygosity			
			Homozygote	Heterozygote	Chimera	WT or Mutations
*ALC*	*alc*	26	0	1(3.85%)	1(3.85%)	24(92.31%)

### Segregation and Genetic Analysis of HDR-mediated Repaired Heterozygote in T_1_ Generation

Because none repaired homozygote was detected in HDR-mediated replacement T_0_ plants, the further segregation of heterozygote was required to generate the anticipant homozygous *alc* allele. The above-mentioned heterozygote plant was selected, which carrying a single copy *Cas9* and *alc* gene (*Cas9*/*-*ALC*/*alc*) was confirmed by Real-time RCR Quantitative detection (Supplementary Table 2). The seeds from this plant were harvested and sowed in greenhouse to generate total of 233 T_1_ plants by strict self-pollination, then 200 random selection plants from them were selected for detection the segregation of *Cas9*/* and *ALC*/*alc* allele respectively. For the former, about 50% (103/200) progenies were heterozygous, and the rest of 25% (49/200) and 25% (48/200) progenies were homozygous *Cas9* gene (*Cas9*/*Cas9*) and homozygous without *Cas9* gene (*/*) respectively. For the latter, the results were basically conformed to the former, about 50% (97/200) plants for the *ALC/alc* allele, 25% (52/200) plants for homozygous *ALC* gene (*ALC*/*ALC*), and 25% (51/200) for the homozygous *alc* gene (*alc*/*alc*). The segregation ratio of descendants was exhibited to be consistent with the expected 1:2:1 segregation ratio of Mendelian Law. Moreover, there was an important finding to note that, among 51 plants of homozygous *alc* allele, there were 11 plants without exogenous *Cas9* gene, in other words, their genotype was (*/*-*alc*/*alc*) (Fig. 1E, Table 3). Ultimately, those plants with the homozygous recessive *alc* gene and Cas9-free were the most desirable for us, and we achieved the purpose of the trait improvement rapidly in tomato.

**Table 3:** Gene segregation in tomato T_1_ generation plants.

Genotype	*Cas9*/*Cas9*	*Cas9*/*	*/*	Total
Genotype				
*ALC*/*ALC*	12	26	14	52
*ALC*/*alc*	24	50	23	97
*alc*/*alc*	13	27	11	51
Total	49	103	48	200

### Off-target effect analysis

The main concern of CRISPR/Cas9 technology is the binding of the nuclease to unintended genomic sites and can cause endogenous genome disruption, which was designated off-target effect. Although low-frequency of off-target effect have been reported in plants^18–20^, to detect the potential off-target events in our experiments, the potential sequences which highly homozygous to the core sequence (20-bp sgRNA and 3-bp PAM) were used as alignments search in the tomato sequencing (http://solgenomics.net/) by BLAST. Total of three putative off-target sites were found which locate on chromosome 1, 2 and 12 respectively (Fig. 1F), and selected as candidates to design specific primers, 30 plants from T_0_ and T_1_ transgenic plants were randomly selected for examination, genomic DNA was extracted from the leaves of each tested plant, and then the putative off-target sites were amplified using PCR with specific designed primers (Supplementary Fig. 5 and Supplementary Table 3). The result showed that no mutations were detected in the supposed off-target sites in all tested plants, thus manifesting that off-target effect wasn’t occurred during mutagenesis and DSBs induced by CRISPR/Cas9 system.

### Characterization of main agronomic traits of HDR-mediated repaired homozygous lines

**Figure 2:**
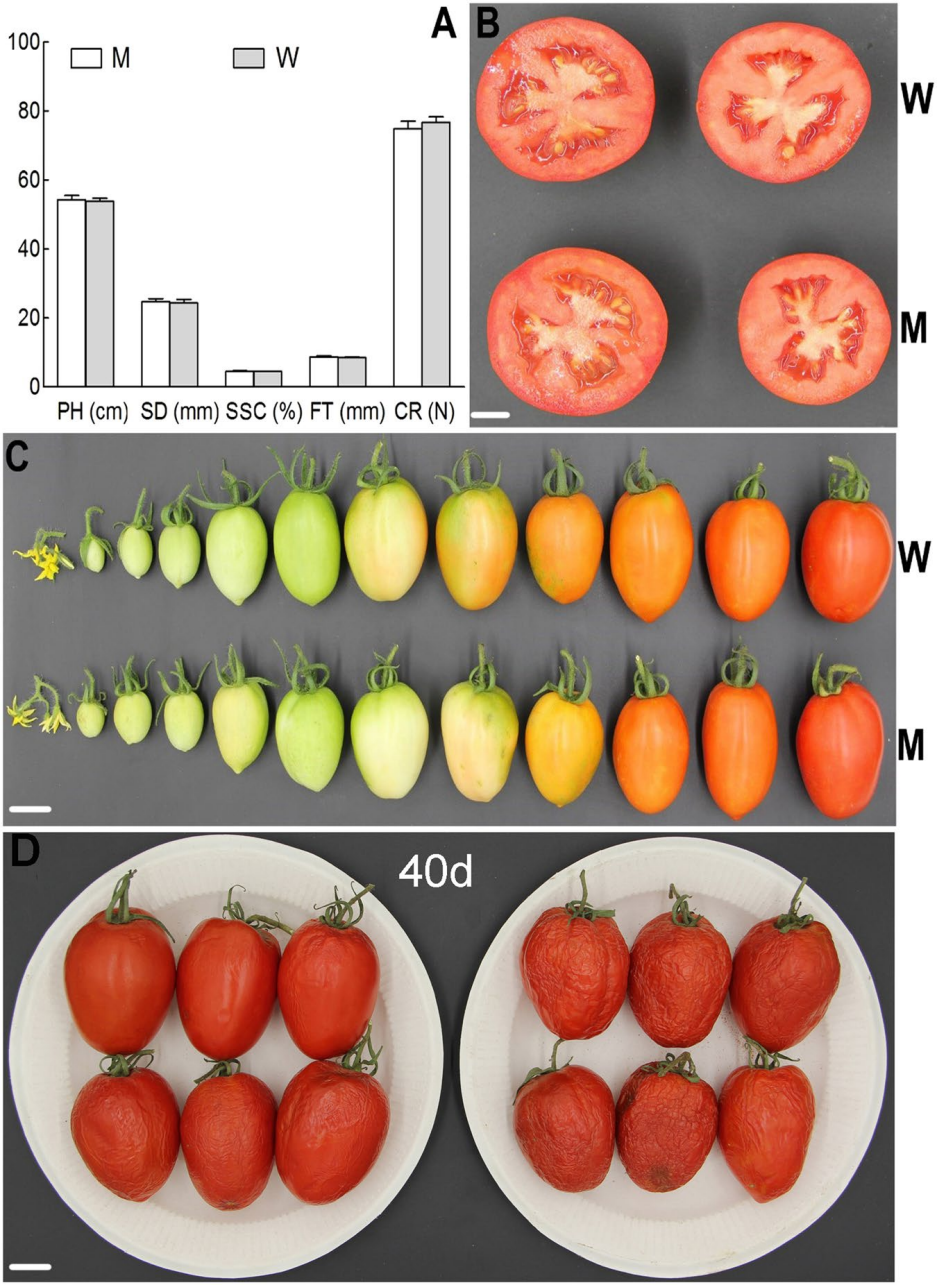
Phenotypic detection of wild-type fruits and mutant fruits. (**A**) Phenotype Value: ‘PH’ means plant height, ‘SD’ means stem diameter, ‘SSC’ means soluble solid content, ‘FT’ means flesh thickness, ‘CR’ means compression resistance, ‘M’ means mutation, ‘W’ means wild-type. (**B**) Comparison of bisected fruit at the ripening stage. (**C**) Developmental series of wild-type fruits (up) and mutant fruits (down). (**D**) Comparison of fruits after 40 days of storage at room temperature, left is homozygous T_1_ mutants and right is wild-type. Bars:1 cm.

To ascertain whether the replacement in the *ALC* gene affect the main agronomic trait, 5 homozygous T_1_ lines randomly selected from the 11 homozygous recessive *alc* gene and Cas9-free lines (*/*- *alc*/*alc*), and 5 wild-type lines (*/*- *ALC*/*ALC*) served as control (CK). The characterization of main agronomic traits was measured by their plant height (PH), stem diameter (SD), fruit soluble solids content (SSC), flash thickness (FT) and fruit firmness (FF), and Student’s t-test revealed that there is no significant difference between them (Fig. 2A and Supplementary Table 4), and other traits were also no difference under normal growth conditions based on observation.

### Dynamic variation pattern of the fruit development and storage

We also investigated the fruit development situation under normal conditions, there were no difference from flowering to fruit enlargement period between them. While from turning color period to the beginning of ripping stage, the fruit of treatment group much delayed than the control group, but both of them could reach maturity at same time (Fig. 2C). These results indicated that *alc/alc* gene in tomato fruit could delay color turning and early ripe, but does not affect the fruit ripening and the harvest time, also does not affect the color of the fruit is ripe (Fig. 2B).

To determine whether replacement in the *ALC* gene affect the character of fruit storage, 6 homozygous T_1_ lines as 6 biological replications was randomly selected from the 11 homozygous recessive *alc* gene. There were 6 mutant lines also do not have Cas9 gene (*/*- alc/alc), and 6 wild-type lines (*/*- ALC/ALC) served as control (CK). One fruit was picked from each line, totally 12 red fruits of homozygous T_1_ mutants and wild-type were analyzed after 40 d stored at room temperature. As seen in photographs taken after 40 d (Fig. 2D), the red fruit of homozygous T_1_ mutant showed only mild dehydration and fruit epidermis appeared wrinkly, in contrast, the red fruit of wild-type exhibited severe dehydration, fruit epidermis appeared wrinkly and began to rot. The results show that *alc/alc* gene can significantly improve the storage time of tomato, and prolong the shelf life.

## Discussion

### Mutation efficiency and genotypes in T_0_ transgenic plants

The experiment results showed that the mutation rate is 72.73% in the T_0_ transgenic lines (Table 1), the data indicated that the CRISPR/Cas9 system efficiently induced DSB in the *ALC* locus with high efficiency of mutagenesis. The result is higher than the previous report in tomato that the average mutation rate was 48%^21^, but lower than that in another report with 83.56%^22^. We speculate the reason would be that the same genotype (M82) was used as material in our and the former experiment, which different from that in the latter experiment (Micro-Tom). Also, the target sequences with low or very high GC contents tend to be edited with poor efficiencies, and a target sequence with GC contents of 50–75% and fewer “GG”s should be selected with priority, we speculate the target sequences with low GC content (45%) is one of the reasons resulted in low off-target. There are many other factors influence mutation rate, including the species, tissue type, the sequence, location and conformation of the target region, also the promoter type for Cas9 driving and the kind of vector system for editing, such as using the separated vector or promoter in expression of Cas9 and gRNA. In addition, there is higher efficiency of obtaining mutagenesis when increasing the number of gRNAs in protoplast with the Csy4 and tRNA expression systems^23^. The available data from the two articles published in the same issue of the journal display that mutation rate was much higher in tobacco and rice protoplasts than in Arabidopsis protoplasts^18,24^. In this study, we used the constitutive promoter *35S* to drive Cas9 and the *Agrobacterium*-mediated transformation platform to deliver the cassette and gained the high efficient of mutations probably because the tomato *ALC* gene could form DSBs easily by CRISPR and prevent off-target sufficiently, even a single gRNA was used. Recent studies revealed that the mutation rate was much higher using the organ specific promoter instead of *35S* promoter to drive Cas9. Such as, the egg cell-specific promoters^25^, the germ-line-specific promoters^26^, the meristem-specific promoter^27^, and the promoter of the *YAO* gene which was preferentially expressed in the embryo sac, the embryo, endosperm and pollen^28^, even synthetic RNA polymerase III promoters^29^ and the Csy4 expression system^23^ to drive the gRNAs. Furthermore, using *Agrobacterium*-mediated transformation or biolistic-dependent transformation, the mutation frequency of former was much higher than the latter^25^.

Most often, four genotypes (homozygote, biallele, heterozygote and chimera) are generated in T_0_ lines. From the perspective of crop breeding, the quickly getting homozygous mutant is an anticipated capability need for trait deployment. However, from our results that we obtained those three genotypes, except the homozygote ones. A total of 26 T_0_ independent lines were generated, and most of them were wt/mutations (92.31%), a few were heterozygote (3.85%) and chimeras (3.85%), but none homozygous mutant was detected from T_0_ plants in our experiment, which were wildly inconsistent with other study results in tomato^21,22^. We speculate the reason why the allele efficiency relatively high was probably due to the difference of genotypes and the target loci, or error-mutation detection. However, most research findings in other crops showed that those were no homozygous mutant in T_0_ plant so far, including two studies in rice^19,30^, All the mutation varieties we obtained were 1- to 2-bp insertions accompany with 1- to 3-bp deletions or numerous-bp deletions, which confirmed the mutagenesis is took place randomly.

### Gene Targeting efficiency through HDR in T_0_ transgenic plants

The repair mechanisms such as NHEJ and HR happen after the production of DNA DSBs. The NHEJ repair system easily and directly connects the broken ends with insertion and/or deletion mutations at high frequency^31^. When DNA donor template was supplied and leaded gene replacement through HDR based repair, namely gene targeting (GT) occurred and repaired the break. Initially, the undesired gene replacement by HDR (GT) was detected when we supplied a double-stranded DNA donor with flanked by two short homology arms (a 100-bp left and a 100-bp right homology arm; data not show). Subsequently, the intended gene substitutions were obtained after we redesigned the dsDNA donor with flanked by two much longer homology arms (a 235-bp left and a 450-bp right homology arm). This result hints that the homology arms of the donor should be enough length for the success of gene replacement through HDR. Even in some studies which designed much longer homolog arms for insert or replace target genes. In the study of tomato *ANT1* overexpression by inserting *35S* promoter and NPTII with 987 bp and 719 bp homolog arms through HDR^32^.

### The strategies to improve the gene targeting efficiency

Although, a single gRNA with different promoter from Cas9 is likely to induce accurately repairing of DSBs as we did in our transform system. In spite of our experiment realized the gene substitution through HDR, the GT efficiency is still on the low side (7.69%). According to the review that was summarized^33^, we analysis it is possible that the following two causes lead to this result. Firstly, the high frequency of NHEJ would suppress the GT efficiency. There is competition between HDR and NHEJ during the resolution of DSBs, and NHEJ is by far the preferred mechanism to repair DNA breaks in higher organisms such as plants. Thus the researchers thought at first for the improvement of GT by means of increasing the efficiency of HDR and suppressing NHEJ. For example, in contrast with the nuclease, the CRISPR/Cas nickase not induce NHEJ-mediated targeted mutagenesis, it could be used efficiently to induce HDR in Arabidopsis^2^. In mammalian systems, identified two small molecules (L755507 and Brefeldin A) that can enhance CRISPR-mediated GT efficiency, 2-3 fold for large fragment insertions and 9-fold for point mutations^34^. Nearly-simultaneously, increased the GT efficiency up to 19-fold by using the inhibitor Scr7 to suppress the DNA ligase IV (a key enzyme in the NHEJ pathway)^35^. Secondly, the *Agrobacterium*-mediated transformation was used to deliver Cas9 and donor template, this method could not feed the cells with sufficient amounts of DNA repair templates to boost the HDR efficiency. Although the GT efficiency is low (7.69%), but it was acceptable from the point of view of crop breeding. However, we still need to find ways for further improving the GT efficiency. It is reported that single-stranded DNA oligonucleotides (ssODN) might produce higher HDR than double strand donor DNA molecule^36^. Although the limited application of those approaches for plant species currently, they may provide some referential ideas or potential solutions to enhance the efficiency of HDR. As the researches move along, many new methods for improving the GT efficiency have been proposed, such as reconstructing Cas9 expression cassettes, introducing paired Cas9 nickases^23^ and a structurally optimized sgRNA^37^, increasing the availability of donor DNA template in target cells^32^. Since only one bases were replaced in our experiment (A → T), the point mutation correction could be inspired by a new approach, which fused a cytidine deaminase enzyme with CRISPR/Cas9. This ‘base editors’ enables the direct conversion of cytidine to uridine, thereby effecting a C → T (or G → A) substitution, with higher GT efficiency without requiring DSBs or a donor template^38^.

### The possible reasons for none homozygous substitution was detected in T_0_ and T_1_ transgenic lines

Getting homozygous mutants quickly is desired by the crop breeders. But there were none homozygous mutation was detected in T_0_ transgenic plants in our experiments. We speculate one reason may be that the cauliflower mosaic virus *35S* promoter was used to drive the expression of Cas9, according to the previous report has indicated that the *35S* promoter has very low activity in germline cells, the constructs with this promoter trend to produce low efficiencies of mutagenesis and generate mosaic plants due to the activity of the Cas9 endonuclease after the first embryogenic cell division^39^. The second reason may be the error detection of homozygous mutant causes the GT efficiency was very low. Also, it could be the donor template and the gRNA are in the same vector which produce the lower proportion of templates and lead to the low of HDR efficiency. In addition, the different transformation tissue probably another reason that we did not gain the homozygous mutant in T_0_ generation.

### Generation of homozygous *alc* elite breeding lines in T_1_ transgenic plants

The heritability and segregation of mutation lines are also analyzed in our study. The merit of CRISPR/Cas9 system is that their components inserted in the genome could be eliminated via selfing or backcrossing. We generated the transgene-free of T_1_ homozygotes HDR-mutants after T-DNA detection by site-specific genomic PCR based Sanger sequencing, which confirms the previous research reports that the transgene region (Exogenous gene) can be easily segregated out in progeny via simple self-fertilization^40^. Combining with the result that these is no off-target mutations were indeed, we preliminary determinate those T_1_ homozygotes HDR-mutants with no trace a transgene are “transgene clean”, and potentially could be used for the breeding program, according to the proposed regulatory framework for genome-edited crops^41^, or the revision of advanced and appropriate regulation in the near future^42,43^. Recently, the first CRISPR-edited organism (a mushroom) received a green light from the US Department of Agriculture (USDA), which gives the confidence for plant biologists that the gene-edited crops falling outside of regulatory authority^44^. Only a few studies have employed the CRISPR/Cas9 system through *Agrobacterium*-transformation for targeted gene modifications in tomato, and all of the studies focus on mutants’ creation^21,22,45,46^. To our knowledge, this is the first study of allelic gene substitution in the tomato based on HDR through *Agrobacterium*-transformation of CRISPR/Cas9 system. Taken together, our results of this study invigorate the potential usefulness of CRISPR/Cas9 gene editing technology in modern crop breeding practices in the near future.

### Generation of new research platform for gene functional study

Quite unexpectedly, we generated Cas9-overexpressing (Cas9-OE) *S*. *lycopersicum* vs M82 transgenic lines from T_1_ segregation population. The Cas9 clone was validated by Sanger sequencing and the expression levels of the Cas9 transcript and protein were confirmed by the analyzing. The capabilities of CRISPR/Cas9 technology giving us the ability to interrogate almost any gene we want, but the transgenic procedures such as stable transformation and tissue culture are more laborious and time-consuming. The Cas9-OE transgenic lines could be used as the foundation for construction of the “virus-mediated genome editing system” (using an optimized the TRV RNA2 genome-derived vector for gRNA delivery), which could be circumvented the tedious procedures of transgenics of each predetermined target sequence, thus this system could be developed as a versatile platform for facilitate gene functional studies in tomato^47^. The constructs of TRV RNA2 contained the gRNA to delivery and permitted the expression of the gRNA were successfully generated in our hands (data not show).

## Methods

### CRISPR/Cas9 Vector Construction

The spCas9 was obtained from Prof. Cai-xia Gao (Chinese Academy of Science, Beijing, China). The sgRNA was designed through the target gene directly and synthetized by Biological Company of Kang Wei, Beijing. The main vector used for the combination is pCAM1301 which stored by our lab. The vector for detecting the ALC gene was repaired by NHEJ or HDR and then constructed using the Circular Polymerase Extension Cloning (CPEC) respectively^48^. The spCas9 was driving by *35* *S* promoter and sgRNA was under the control of the aribidopsis (*Aribidopsis thaliana*) U6 promoter. The pCAM1301 binary vector constructed: *35S*-Cas9-AtU6-sgRNA and this vector used for observing the NHEJ repair. As for the HDR repair, another pCAM1301 vector connected with a double-strand DNA donor repair template which designed based on the target site sequence of *ALC* gene. This ds-donor has 235-bp sequence was homologous to the left side of the target site in *SLALC*, while the 450-bp sequence was homologous to the right side of the target site. First, these two constructed vector were cloned into the bacteria of DH5a using the Heat shock transformation. After sequencing the accuracy of constructed vector, they were transformed into *A*. *tumefaciens* EHA105 by the Liquid nitrogen freezing and thawing.

### *Agrobacterium tumefaciens*-Mediated Transformation

Transformation of gene mutagenesis and gene targeting through HDR were performed in the M82 WT genetic background. *A*. *tumefaciens*-mediated transformations were performed according to previous study^49^. In brief, tomato seeds were germinated on ½ MS medium after sterilization with 20% NaClO. After 14–16 days culture, the apical segments of hypocotyls were punctured with OD_600_ = 1.0 of *Agrobacterium* suspension. For the NHEJ repair the *Agrobacterium* suspension which have *35S*-Cas9-AtU6-sgRNA vector. For the HDR repair the *Agrobacterium* suspension which have the same concentration of *35S*-Cas9-AtU6-sgRNA and ds-donor template vector respectively. Then, the explants were inoculated on selective plates with hygromycin (3 mg/L) until transgenic plants were regenerated from the calluses. After rooting, the regenerated transgenic plants were moved to a light growth chamber.

### Transgenic Lines Assay and Genotyping

Genomic DNA from tomato T_0_ transgenic lines was extracted using CTAB methods, and the genomic flanks containing the target sites were amplified using specific primers. Then, the annealed PCR products were subjected in to 1% agarose. The cut and purified PCR products were cloned into the pZERO-T Vector (Transgen, China), and 24 clones for mutagenesis and 10 clones for targeted gene replacement were sequenced at each plant with the M13 primer, for genotyping of T_1_ plants which obtained from the T_0_ lines by strict self-pollination, the target fragments were directly sequenced.

### Off-target effect analysis

According to the sequencing data of tomato from Connell, the off-target sites were Blastn analyzed by blasting 20 bp target site and 3 bp PAM sequence. Total 3 most likely off-target sites containing fewer than 7-bp mismatches in the 20-bp seed sequence were selected and used to design specific primers. The genomic DNA surrounding the potential off-target sites was amplified using specific primers PCR products were analyzed by Sanger sequencing.

### Characterization of agronomic traits, dynamic variation pattern of the fruit development and storage

Wild-type and homozygous T_1_ mutant lines were grown in the field under normal growth conditions in Urumqi. Agronomic traits were characterized by measuring plant height (PH), stem diameter (SD), fruit soluble solids content (SSC), flash thickness (FT) and fruit firmness (FF). Ten plants were investigated for each line. To detect the dynamic variation pattern of the fruit storage, red fruits of homozygous T_1_ mutants and wild-type were detached, stored at room temperature ranging from 20 to 25 °C with nearly 40% relative humidity, and compared and analyzed after 40 days.

## Electronic supplementary material

Supplementary information

## References

[1] Tester M, Langridge P (2010). Breeding Technologies to Increase Crop Production in a Changing World. Science (80-.).

[2] Fauser F, Schiml S, Puchta H (2014). Both CRISPR/Cas-based nucleases and nickases can be used efficiently for genome engineering in Arabidopsis thaliana. Plant J..

[3] Belhaj K, Chaparro-Garcia A, Kamoun S, Patron NJ, Nekrasov V (2015). Editing plant genomes with CRISPR/Cas9. Curr. Opin. Biotechnol..

[4] Puchta H, Fauser F (2014). Synthetic nucleases for genome engineering in plants: Prospects for a bright future. Plant Journal.

[5] Rick CM, Butler L (1956). Cytogenetics of the Tomato. Adv. Genet..

[6] Garg N, Cheema DS, Dhatt AS (2008). Genetics of yield, quality and shelf life characteristics in tomato under normal and late planting conditions. Euphytica.

[7] Saladié M (2007). A reevaluation of the key factors that influence tomato fruit softening and integrity. Plant Physiol..

[8] Casals J (2012). Genetic basis of long shelf life and variability into Penjar tomato. Genet. Resour. Crop Evol..

[9] Bota J (2014). Characterization of a landrace collection for Tomatiga de Ramellet (Solanum lycopersicum L.) from the Balearic Islands. Genet. Resour. Crop Evol..

[10] Robinson RW TML (1968). Ripening inhibitor: a gene with multiple effects on ripening. Tomato Genet Coop Rep.

[11] Tigchelaar ECM, Tomes ML, Kerr EA BRJ (1973). A new fruit ripening mutant, non-ripening (nor). Tomato Genet Coop Rep.

[12] Thompson AJ (1999). Molecular and genetic characterization of a novel pleiotropic tomato-ripening mutant. Plant Physiol.

[13] Paran I, Van Der Knaap E (2007). Genetic and molecular regulation of fruit and plant domestication traits in tomato and pepper. Journal of Experimental Botany.

[14] Garg N, Cheema DS (2008). Genotype environment interactions for shelf life and yield attributes in tomato hybrids heterozygous at rin, nor, or alc loci. J. Crop Improv..

[15] McGlasson WB, Last JH, Shaw KJ, Meldrum SK (1987). Influence of the non-ripening mutants rin and nor on the aroma of tomato fruit. HortScience.

[16] Kopeliovitch E, Mizrahi Y, Rabinowitch HD, Kedar N (1982). Effect of the fruit-ripening mutant genes rin and nor on the flavor of tomato fruit. J. Am. Soc. Hortic. Sci..

[17] Casals J (2011). Long-term postharvest aroma evolution of tomatoes with the alcobaça (alc) mutation. Eur. Food Res. Technol..

[18] Shan Q (2013). Targeted genome modification of crop plants using a CRISPR-Cas system. Nat. Biotechnol..

[19] Zhang H (2014). The CRISPR/Cas9 system produces specific and homozygous targeted gene editing in rice in one generation. Plant Biotechnol. J..

[20] Sun X (2015). Targeted mutagenesis in soybean using the CRISPR-Cas9 system. Sci. Rep..

[21] Brooks C, Nekrasov V, Lippman ZB, Van Eck J (2014). Efficient Gene Editing in Tomato in the First Generation Using the Clustered Regularly Interspaced Short Palindromic Repeats/CRISPR-Associated9 System1. Plant Physiol.

[22] Pan C (2016). CRISPR/Cas9-mediated efficient and heritable targeted mutagenesis in tomato plants in the first and later generations. Sci. Rep..

[23] Cermak T (2017). A Multipurpose Toolkit to Enable Advanced Genome Engineering in Plants. Plant Cell.

[24] Li J-F (2013). Multiplex and homologous recombination-mediated genome editing in Arabidopsis and Nicotiana benthamiana using guide RNA and Cas9. Nat. Biotechnol..

[25] Wang Z-P (2015). Egg cell-specific promoter-controlled CRISPR/Cas9 efficiently generates homozygous mutants for multiple target genes in Arabidopsis in a single generation. Genome Biol..

[26] Mao Y (2016). Development of germ-line-specific CRISPR-Cas9 systems to improve the production of heritable gene modifications in Arabidopsis. Plant Biotechnol. J..

[27] Hyun Y (2015). Site-directed mutagenesis in Arabidopsis thaliana using dividing tissue-targeted RGEN of the CRISPR/Cas system to generate heritable null alleles. Planta.

[28] Yan L (2015). High-Efficiency Genome Editing in Arabidopsis Using YAO Promoter-Driven CRISPR/Cas9 System. Molecular Plant.

[29] Schwartz CM, Hussain MS, Blenner M, Wheeldon I (2016). Synthetic RNA polymerase III promoters facilitate high efficiency CRISPR-Cas9 mediated genome editing in Yarrowia lipolytica. ACS Synth. Biol..

[30] Ma X (2015). A Robust CRISPR/Cas9 System for Convenient, High-Efficiency Multiplex Genome Editing in Monocot and Dicot Plants. Mol. Plant.

[31] Hisano Y (2015). Precise in-frame integration of exogenous DNA mediated by CRISPR/Cas9 system in zebrafish. Sci. Rep..

[32] Čermák T, Baltes NJ, Čegan R, Zhang Y, Voytas DF (2015). High-frequency, precise modification of the tomato genome. Genome Biol..

[33] Zhang D, Li Z, Li JF (2016). Targeted Gene Manipulation in Plants Using the CRISPR/Cas Technology. Journal of Genetics and Genomics.

[34] Yu C (2015). Small molecules enhance crispr genome editing in pluripotent stem cells. Cell Stem Cell.

[35] Maruyama T (2015). Increasing the efficiency of precise genome editing with CRISPR-Cas9 by inhibition of nonhomologous end joining. Nat. Biotechnol..

[36] Chen F (2011). High-frequency genome editing using ssDNA oligonucleotides with zinc-finger nucleases. Nat. Methods.

[37] Zhao P (2016). One-step homozygosity in precise gene editing by an improved CRISPR/Cas9 system. Cell Res..

[38] Komor AC, Kim YB, Packer MS, Zuris JA, Liu DR (2016). Programmable editing of a target base in genomic DNA without double-stranded DNA cleavage. Nature.

[39] Eid A, Ali Z, Mahfouz MM (2016). High efficiency of targeted mutagenesis in arabidopsis via meiotic promoter-driven expression of Cas9 endonuclease. Plant Cell Rep..

[40] Xu R-F (2015). Generation of inheritable and ‘transgene clean’ targeted genome-modified rice in later generations using the CRISPR/Cas9 system. Sci. Rep..

[41] Huang S, Weigel D, Beachy RN, Li J (2016). A proposed regulatory framework for genome-edited crops. Nat. Genet..

[42] Ishii T, Araki M (2016). Consumer acceptance of food crops developed by genome editing. Plant Cell Rep..

[43] Jones HD (2015). Future of breeding by genome editing is in the hands of regulators. GM Crops Food.

[44] Waltz E (2016). Gene-edited CRISPR mushroom escapes US regulation. Nature.

[45] Ron M (2014). Hairy root transformation using Agrobacterium rhizogenes as a tool for exploring cell type-specific gene expression and function using tomato as a model. Plant Physiol..

[46] Ito Y, Nishizawa-Yokoi A, Endo M, Mikami M, Toki S (2015). CRISPR/Cas9-mediated mutagenesis of the RIN locus that regulates tomato fruit ripening. Biochem. Biophys. Res. Commun.

[47] Ali Z (2015). CRISPR/Cas9-mediated viral interference in plants. Genome Biol..

[48] Tian JQJ, Quan J (2009). Circular Polymerase Extension Cloning of Complex Gene Libraries and Pathways. PLoS One.

[49] Sivankalyani V, Takumi S, Thangasamy S, Ashakiran K, Girija S (2014). Punctured-hypocotyl method for high-efficient transformation and adventitious shoot regeneration of tomato. Sci. Hortic. (Amsterdam).

