# Supplementary material for: CRISPR/Cas9-induced Targeted Mutagenesis and Gene Replacement to Generate Long-shelf Life Tomato Lines
Source: Sci Rep. 2017 Sep 19;7:11874. doi: 10.1038/s41598-017-12262-1 (PMC5605656; doi:10.1038/s41598-017-12262-1)
Supplement: Supplementary file 1 — Supplementary information [file 41598_2017_12262_MOESM1_ESM.doc]

**Supplementary information**

**CRISPR/Cas9-induced Targeted Mutagenesis and Gene Replacement to Generate Long-shelf Life Tomato Lines**

**Qing-hui Yu^1*^, Baike Wang^1,2^, Ning Li^1,2^, Yaping Tang^1,2^, Shengbao Yang^1,2^, Tao Yang^1^, Juan Xu****^1^, Chunmiao Guo^1^, Peng Yan^1^, Qiang Wang^1^ and Patiguli Asmutola^1^**

^1^Institute of Horticulture, Xinjiang Academy of Agricultural Science, Urumqi 830091, China.

^2^These authors contributed equally to the article.

*Address correspondence to [yuqinghui98@sina.com](mailto:yuqinghui98@sina.com)

Summary

Inducing targeted gene replacement using CRISPR/Cas9 system via homology-directed repair (HDR) to generate tomato long-shelf life breeding elites in T_1_ generations.

Author contributions:

Q.H.Y., B.W., N.L., Y.T., and S.Y. conceived and designed the experiments; B.W., N.L., Y.T., and S.Y. performed the experiments; T.Y., C.G., P.Y., J.X., P.A., and Q.W. analyzed the data; Q.H.Y. wrote the article with contribution of all the authors; Q.H.Y. supervised and complemented the writing.

Funding information:

This work was supported by S&T innovation talents cultivation project for excellent youth of Xinjiang (2014721023), and major project in the “12th Five-year” Plans for S&T of Xinjiang (201230116-3) and special fund for agro-scientific research in the public interest of China (201303115).

Corresponding author:

Qing-hui Yu

[yuqinghui98@sina.com](mailto:yuqinghui98@sina.com)

***Supplementary Table 1. Primers for detection of ALC/ALC target region***

| Target locus | Sequence |
| --- | --- |
| *ALC* Forward  *ALC* Reverse | acaaacataaagtagtggaccca  acctctttcggctatttcgtata |

**Supplementary Table 2. Template values of Cas9 and *ACTIN* genes in samples obtained by quantitative PCR**

| Transgenic lines | Cas9 gene | | ACTIN | | Ratio to ACTIN | 2*ACL /ACTIN | By real-time quantity PCR |
| --- | --- | --- | --- | --- | --- | --- | --- |
|  | Ct value | Amount of  template | Ct  value | Amount of  template |  |  |  |
| 1 | 19. 91 ± 0. 32 | 0.128 | 24. 25 ± 0. 11 | 0.214 | 0.598 | 1.196 | 1 |

**Supplementary Table 3. Primers for detection of off-target**

| Target site ID | Target Site sequence | Oligo-F (5’-3’) | Oligo-R(5’-3’) |
| --- | --- | --- | --- |
| NI1 | GTTGGCCAAAAAGTAAAAACCGG | AAATCCTTACGCCTTCTTCG | TCATAGCCCATTGACTGGC |
| NI2 | GACTCAAATAAAGTAAAAACCGG | CCACCCTTTGGAGATTCTTC | TCGGTTGCTTTCTTTGTCAC |
| NI3 | ATCAAATACGAAGTAAAAACCGG | TTCTGATGTTGCCAGCCA | CGTTAGTTTCTTGGATGCCC |

**Note:** Red fonts are the mismatch nucleotides, blued fonts are the PAM sequence.

**Supplementary Tables 4. The variance analysis of phenotypic data**

| Sources of variance | Dependent variable | F | Sig. |
| --- | --- | --- | --- |
| Corrected Model | plant height(cm) | 0.405 | 0.750 |
|  | stem diameter(mm) | 0.087 | 0.967 |
|  | soluble solid content(%) | 0.319 | 0.811 |
|  | flesh thickness(mm) | 1.056 | 0.385 |
|  | compression resistance(N) | 1.463 | 0.248 |
| Intercept | plant height(cm) | 4662.780 | 0.000 |
|  | stem diameter(mm) | 1142.998 | 0.000 |
|  | soluble solid content(%) | 2885.178 | 0.000 |
|  | flesh thickness(mm) | 3026.385 | 0.000 |
|  | compression resistance(N) | 3350.274 | 0.000 |
| Type | plant height(cm) | 0.064 | 0.803 |
|  | stem diameter(mm) | 0.087 | 0.770 |
|  | soluble solid content(%) | 0.155 | 0.697 |
|  | flesh thickness(mm) | 0.847 | 0.366 |
|  | compression resistance(N) | 0.493 | 0.489 |
| repetition | plant height(cm) | 0.576 | 0.569 |
|  | stem diameter(mm) | 0.086 | 0.917 |
|  | soluble solid content(%) | 0.402 | 0.673 |
|  | flesh thickness(mm) | 1.160 | 0.329 |
|  | compression resistance(N) | 1.948 | 0.163 |


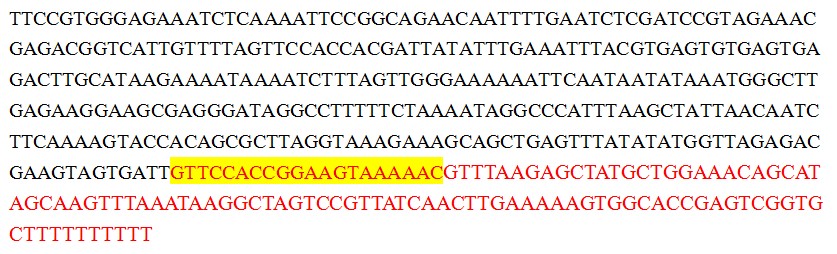


**Supplementary Figure 1.** The sequence of the gRNA expression vector pAtU6-sgRNA. The Arabidopsis U6 promoter is marked in black, and the gRNA scaffold in red. The guide sequence is highlighted in yellow.


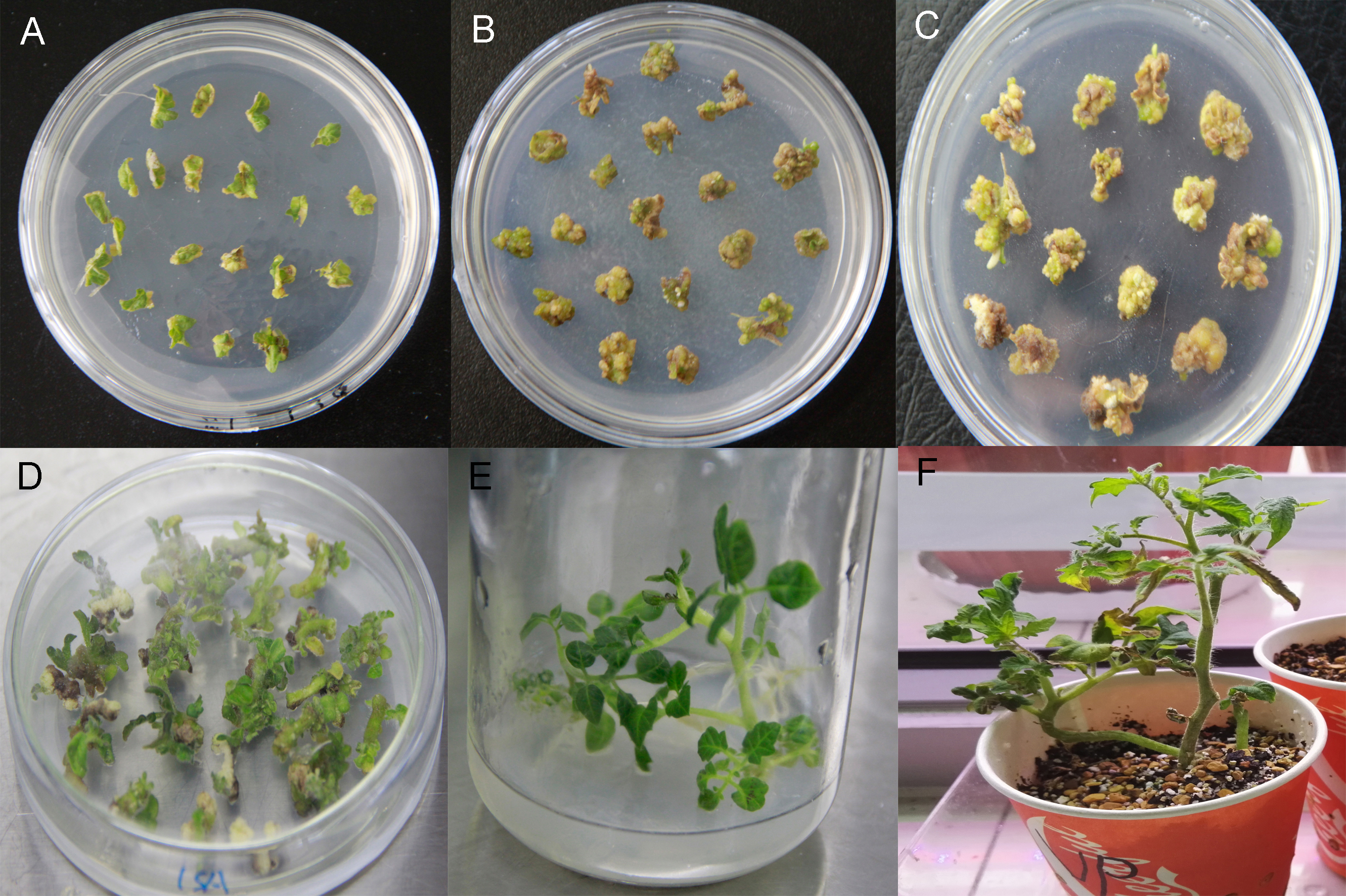


**Supplementary Figure 2.** The culture process of transforming Cas9 gene of Tomato. **A** to **F** is the sequence of culture.


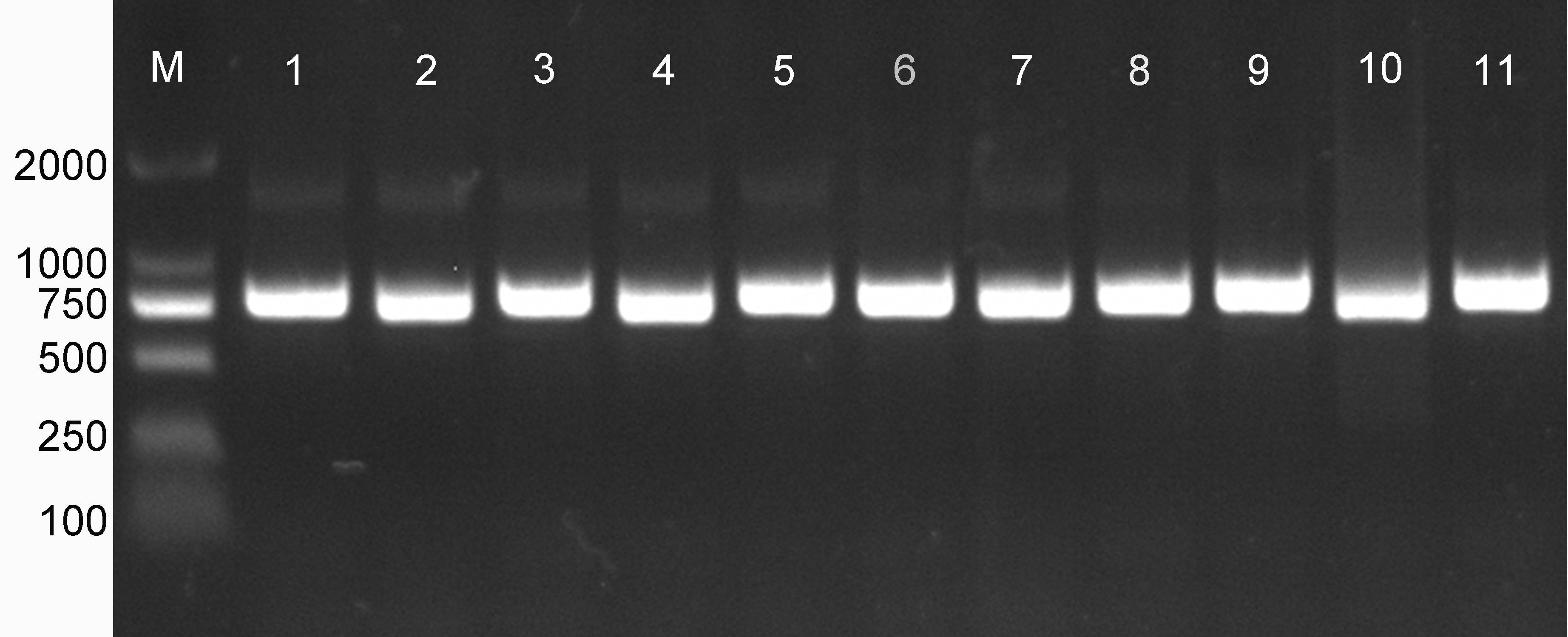


**Supplementary Figure 3.** PCR results of *Alc/alc* target gene. The lane of 1 to 11 is the PCR product of 11 transgenic plants.


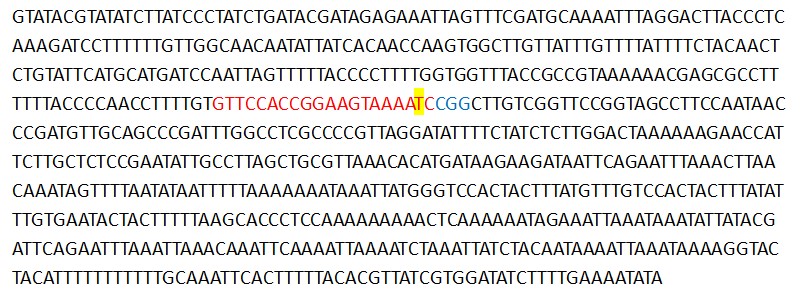


**Supplementary Figure 4.** DNA sequence of the *alc* donor template. The sgRNA site is marked in red. The PAM site is marked in blue. The one nucleotide substitutions is highlighted in yellow.


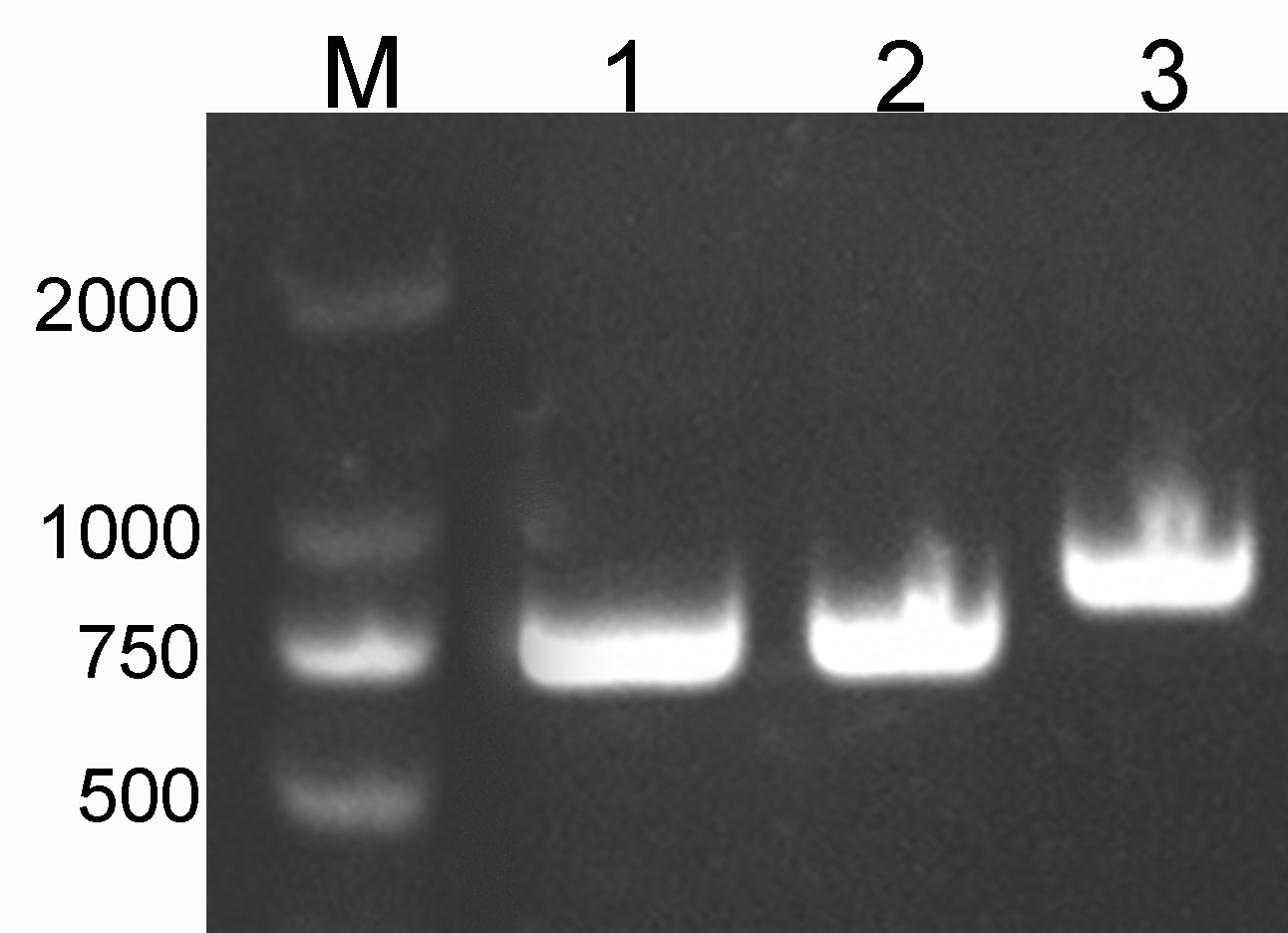


**Supplementary Figure 5**. Off-target verification. The primers from 1 to 3 are NI1, NI2 and NI3 respectively. The PCR results are 750 bp, 750bp and 950 bp separately.
